# Targeted analysis of whole exome sequencing in Thai patients with neonatal diabetes

**DOI:** 10.1007/s00439-025-02815-0

**Published:** 2026-01-07

**Authors:** Nattachet Plengvidhya, Thanida Tangjarusritaratorn, Nipaporn Teerawattanapong, Tassanee Narkdontri, Saranya Innang, Suavaluk Songlilitchuwong, Sarocha Suthon, Watip Tangjittipokin

**Affiliations:** 1 Division of Endocrinology and Metabolism, Department of Medicine, Faculty of Medicine Siriraj Hospital, Mahidol University, Bangkok, Thailand; 2 Department of Immunology, Faculty of Medicine Siriraj Hospital, Mahidol University, Bangkok, 10700 Thailand; 3 Siriraj Center of Research Excellence for Diabetes and Obesity (SiCORE- DO), Research Department, Faculty of Medicine Siriraj Hospital, Mahidol University, Bangkok, Thailand; 4 Department of Research, Faculty of Medicine Siriraj Hospital, Mahidol University, Bangkok, Thailand

**Keywords:** Variant, Neonatal diabetes mellitus, Diabetes, Whole exome sequencing, Genetic resources

## Abstract

Neonatal diabetes mellitus (NDM) typically presents within the first 6 months of life and generally lacks islet autoantibodies. Genetic elucidation of NDM is a prime example of precision medicine in diabetes. However, no published genetic data exist for NDM in Thailand. We aimed to assess the genetic etiology of Thai NDM using whole exome sequencing (WES). We enrolled 14 Thai patients with NDM and measured GAD65, IA-2, and ZnT8 autoantibodies. We then performed WES and analyzed 43 NDM-related genes to identify causative variants. All subjects tested negative for the three islet autoantibodies. Eight harbored variants in well-established NDM genes (*KCNJ11* [*n* = 5], *ABCC8* [*n* = 1], *INS* [*n* = 2 in identical twins]). Two patients with *KCNJ11* variants (rs80356616: p.Val59Met and rs8035661: p.Arg50Gln) achieved excellent glycemic control on sulfonylureas, illustrating precision therapy. The remaining six carried pathogenic variants in genes associated with monogenic diabetes, including *LRBA*, *EIF2AK3*, *DOCK8*, *WFS1*, *GATA6*, *CISD2/SLC9B1*, and *COQ2*. This is the first report on the genetic etiology of NDM in Thailand. WES is an effective approach to identifying variants in this rare diabetes subtype. Larger cohort studies are needed to determine the true prevalence of NDM in Thailand.

**Supplementary Information:**

The online version contains supplementary material available at 10.1007/s00439-025-02815-0.

## Introduction

Neonatal diabetes mellitus (NDM) is a form of monogenic diabetes typically diagnosed within the first 6 months of life. It arises from single-gene variants that impair pancreatic β-cell function. The absence of islet auto-antibodies helps distinguish NDM from type 1 diabetes. Monogenic diabetes accounts for approximately 2.5% to 6.5% of pediatric diabetes (Greeley et al. 2022). The incidence of NDM is estimated at 1 in 90,000 live births in Italy (Iafusco et al. 2012). Transient and permanent types are the two main forms of NDM (Beltrand et al. 2020). In a Thai diabetes registry of patients diagnosed before age 30, the prevalence of NDM was 0.8% (Dejkhamron et al. 2022).

Previous reports identified variants in three common genes—*ABCC8*,* KCNJ11*, and *INS*—associated with NDM (De Franco et al. 2020). *ABCC8* and *KCNJ11* encode subunits of the ATP-sensitive potassium (KATP) channel (SUR1 and KIR6.2, respectively) in pancreatic β-cells. Activating variants in these genes keeps the KATP channels open, leading to persistent membrane hyperpolarization and reduced insulin release, which causes hyperglycemia (Hattersley et al. 2018). Variants in other genes, including *GCK*,* EIF2AK3*,* WFS1*,* RFX6*,* and HNF1B*, are also implicated in NDM and specific clinical presentations (Lemelman et al. 2018). Genetic alterations in KATP channel genes and the *INS* gene have been identified as principal contributors to NDM across multiple populations (Barbetti et al. 2024).

Accurate molecular analysis of NDM is crucial because it guides optimal treatment. In particular, patients with *KCNJ11* or *ABCC8* variants can often switch from insulin to oral sulfonylureas, resulting in a marked and sustained improvement in glycated hemoglobin levels (Pearson et al. 2006). Such cases exemplify precision medicine in diabetes therapy.

Currently, no systematic study in Thailand has addressed the genetic etiology of NDM. The only study conducted focused on genetic causes in Thai T1D patients with negative autoantibodies and an age of onset greater than 1 year (Teerawattanapong et al. 2024). Therefore, this study aimed to determine whether variants in NDM-related genes are associated with diabetes in Thai patients with NDM using next-generation whole exome sequencing (WES). We also examined associations between these variants, clinical presentations, and treatment outcomes.

## Materials and methods

### Patients

Participants with NDM diagnosed before 1 year of age were enrolled in Thailand from May 2019 to December 2022. The study protocol and informed consent were approved by the Faculty of Medicine Siriraj Hospital Ethics Review Board, Mahidol University, Thailand (COA no. Si 491/2014). Informed consent was obtained from all parents of the participants. NDM was diagnosed using the 2022 ISPAD criteria (Greeley et al. 2022), which require: (a) onset before 1 year of age, (b) low or undetectable C-peptide levels, and (c) negative islet autoantibodies.

Fourteen patients were recruited. All 14 enrolled patients were of Thai ethnicity. GAD65, IA-2, and ZnT8 autoantibodies were measured by the RSR ELISA test (RSR, Cardiff, UK). Fasting plasma glucose was measured using an enzymatic (hexokinase) assay, while HbA1c was determined by high-performance liquid chromatography. Creatinine was analyzed via an enzymatic method, and C-peptide was quantified by electrochemiluminescence immunoassay (Roche Diagnostics, Mannheim, Germany). All tests were performed at the Department of Clinical Pathology, Faculty of Medicine Siriraj Hospital, Mahidol University, Bangkok, Thailand.

### Sample Preparation and whole exome sequencing (WES)

Genomic DNA was extracted from buffy coats using the FlexiGene DNA kit (Qiagen, Valencia, CA, USA). Variant analysis of the twin patients (*ABCC8*,* KCNJ11*, and *INS*) was performed via Sanger sequencing at the Exeter Genomic Laboratory, UK. For the remaining twelve patients, WES was conducted at Macrogen Inc. (NGS), Seoul, Korea, using 100 ng of DNA per sample. Library preparation followed the Agilent SureSelect Target Enrichment protocol (SureSelect V7-post), and sequencing was carried out on an Illumina HiSeq 4000 platform (Illumina Inc.).

All sequence reads were aligned to the UCSC human genome reference hg19 (original GRCh37 from NCBI, February 2009) using the Burrows‒Wheeler Alignment Tool version 0.7.12. Variants were called with the Genome Analysis Toolkit version 3.4.0 and annotated using SnpEff version 4.1 g. All variants were cross-referenced with the dbSNP142 annotation database.

### Bioinformatic analysis

**Fig. 1:**
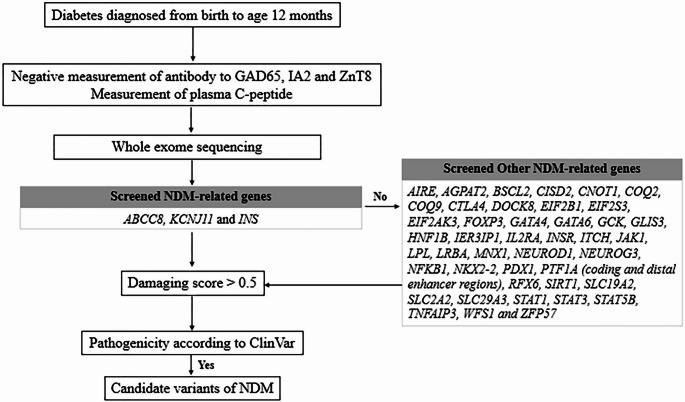
Flowchart of candidate variant analysis in patients with neonatal diabetes mellitus

We first screened for pathogenic variants in the three most common NDM genes (*ABCC8*,* KCNJ11*, and *INS*). If no pathogenic variants were detected, we extended the analysis to the following NDM-related genes: *AIRE*,* AGPAT2*,* BSCL2*,* CISD2*,* CNOT1*,* COQ2*,* COQ9*,* CTLA4*,* DOCK8*,* EIF2B1*,* EIF2S3*,* EIF2AK3*,* FOXP3*,* GATA4*,* GATA6*,* GCK*,* GLIS3*,* HNF1B*,* IER3IP1*,* IL2RA*,* INSR*,* ITCH*,* JAK1*,* LPL*,* LRBA*,* MNX1*,* NEUROD1*,* NEUROG3*,* NFKB1*,* NKX2-2*,* PDX1*,* PTF1A* (coding and distal enhancer regions), *RFX6*,* SIRT1*,* SLC19A2*,* SLC2A2*,* SLC29A3*,* STAT1*,* STAT3*,* STAT5B*,* TNFAIP3*,* WFS1*, and *ZFP57* (Fig. 1). All NDM-related genes were reported in a previous study (De Franco et al. 2015).

Candidate variants were then evaluated using 19 in silico algorithms available in the Varcards platform (http://www.genemed.tech/varcards2) (Wang et al. 2024): SIFT, PolyPhen2 (HDIV, HVAR), LRT, MutationTaster, FATHMM, PROVEAN, MetaSVM, MetaLR, VEST3, M-CAP, CADD, DANN, Fathmm-MKL, Eigen, GenoCanyon, fitCons, REVEL, and REVE. Variants with a damaging score greater than 0.5 across 19 in silico algorithms were considered potentially pathogenic. Finally, we assessed these candidate variants for pathogenicity based on the ClinVar database (https://www.ncbi.nlm.nih.gov/clinvar/). Furthermore, the final classification of variants followed the ACMG guidelines established in 2015 (Richards et al. 2015). Specifically, we have adjusted the application of PM2 (low frequency in 1000 Genomes) and PP3 (Multiple lines of computational evidence support a deleterious effect on the gene conservation and splicing impact) to the supporting level where appropriate.

## Results

### Clinical and biochemical characteristics of NDM

The clinical characteristics and biochemical profiles of 14 NDM patients are presented in Table 1. All patients tested negative for anti-GAD65, anti-IA-2, and anti-ZnT8 antibodies. Most exhibited diabetes as their sole clinical manifestation, although some experienced diabetic ketoacidosis or hypoglycemia. One patient also had developmental dysplasia of the hip.

**Table 1:** Clinical characteristics of enrolled patients with neonatal diabetes mellitus

Patient number	Age (years)	Age at diagnosis (months)	Sex	Clinical presentation	Weight (kg)	Height (cm)	BMI (kg/m^2^)	Autoantibody	C-peptide (ng/ml)	FPG (mg/dl)	HbA1c (%)
015	0.92	11 days	Male	DKA	9.50	73	17.73	*Negative	NA	NA	9.2
039	10	1	Male	Hypoglycemia	16.80	116	12.49	*Negative	NA	60 (CBG)	8.5
040	0.17	2	Male	DKA	5.93	59.80	16.58	*Negative	NA	720 (CBG)	9.8
133	8	6	Female	NA	6.00	71	11.90	**Negative	*0.229	NA	9.3
187	6	1	Male	Developmental dysplasia of the hip	20.80	110	17.19	**Negative	*1.186	NA	5.8
579	9	11	Male	DKA	33.40	143	16.33	**Negative	*0.010	269	6.9
587	0.5	6	Male	NA	5.47	64	13.35	**Negative	*0.058	581	13.1
599	10	Birth	Female	NA	22.00	126	13.86	**Negative	*0.069	NA	NA
600	10	Birth	Female	NA	23.00	125	14.72	**Negative	*0.058	NA	NA
851	20	Birth	Female	NA	56.60	151.5	24.66	**Negative	**0.020	259	9.38
970	1	Birth	Male	NA	1.60*	44*	8.26*	**Negative	**0.480	584	NA
1077	0.17	2	Female	NA	4.00	51.5	15.08	**Negative	**0.600	894	11.1
1342	2	3	Male	NA	5.50	56	17.54	**Negative	**<0.020	210	8.7
1370	29	12	Male	NA	69.10	169	24.19	**Negative	**<0.020	412	7.9

*BMI* body mass index, *CBG* capillary blood glucose, *DKA* diabetic ketoacidosis, *FPG* fasting plasma glucose, *NA* not available

* Weight, height, and BMI were measured at birth unless otherwise noted; if not, they were measured at enrollment

Autoantibody testing: * anti-GAD and IA-2 were measured; ** anti-GAD, IA-2, and ZnT8 were measured

C-peptide assay: * measured by chemiluminescence immunoassay; ** measured by electrochemiluminescence immunoassay

### Whole exome sequencing (WES) results

**Fig. 2:**
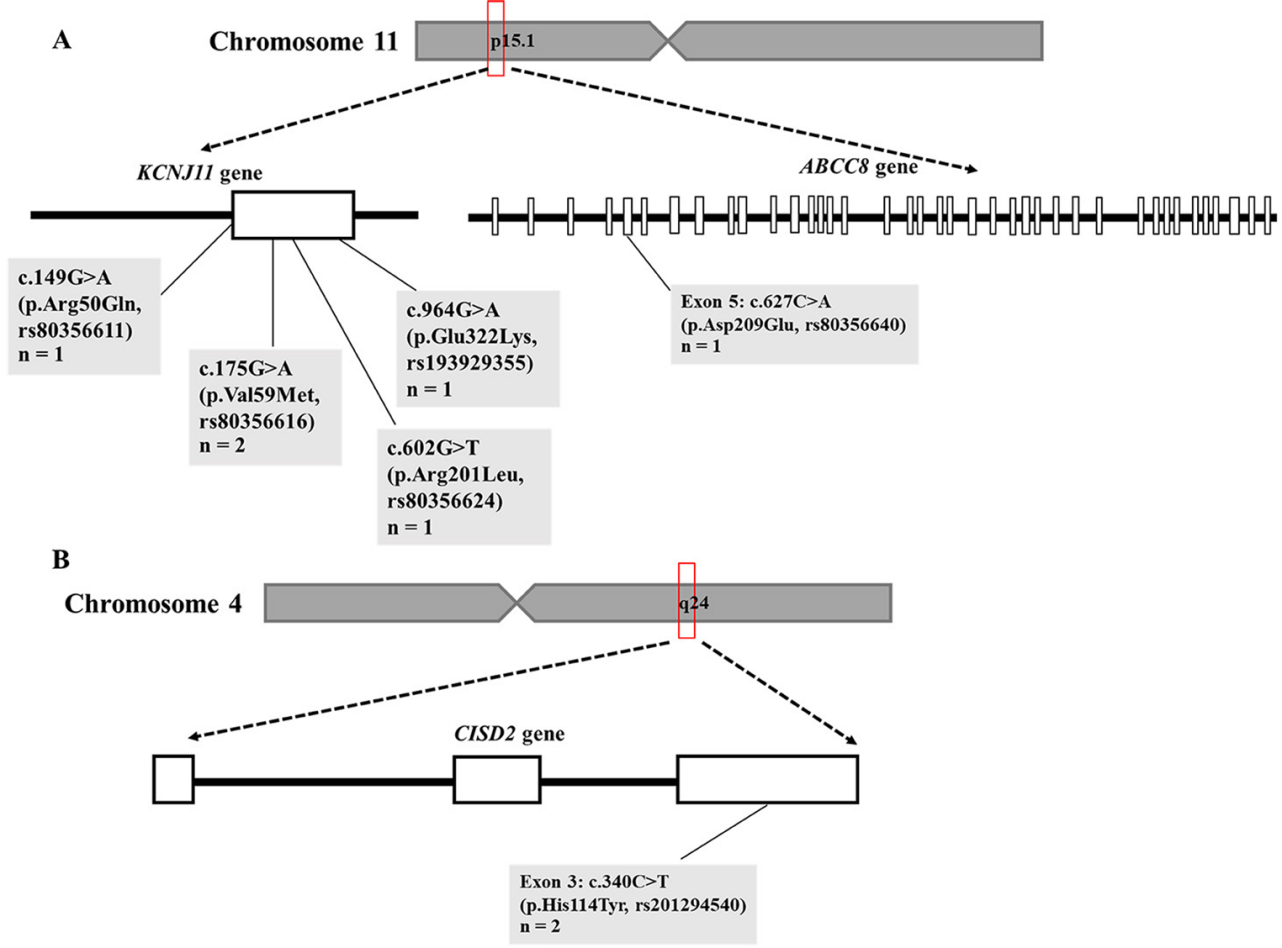
Spectrum of genetic variants in *KCNJ11*,* ABCC8*, and *CISD2* identified in Thai children with neonatal diabetes mellitus. **A** Genetic variants on chromosome 11 at position q15.1 in *KCNJ11*accounted for 36% of 14 cases (c.149G > A, p.Arg50Gln, *n* = 1; c.175G > A, p.Val59Met, *n* = 2; c.602G > T, p.Arg201Leu, *n* = 1; c.964G > A, p.Glu322Lys, *n* = 1), while a variant in exon 5 of *ABCC8* (c.627 C > A, p.Asp209Glu, *n* = 1) was also identified. **B** A genetic variant on chromosome 4 at position q24 in exon 3 of *CISD2* (c.340 C > T, p.His114Tyr) was identified in 2 patients

**Fig. 3:**
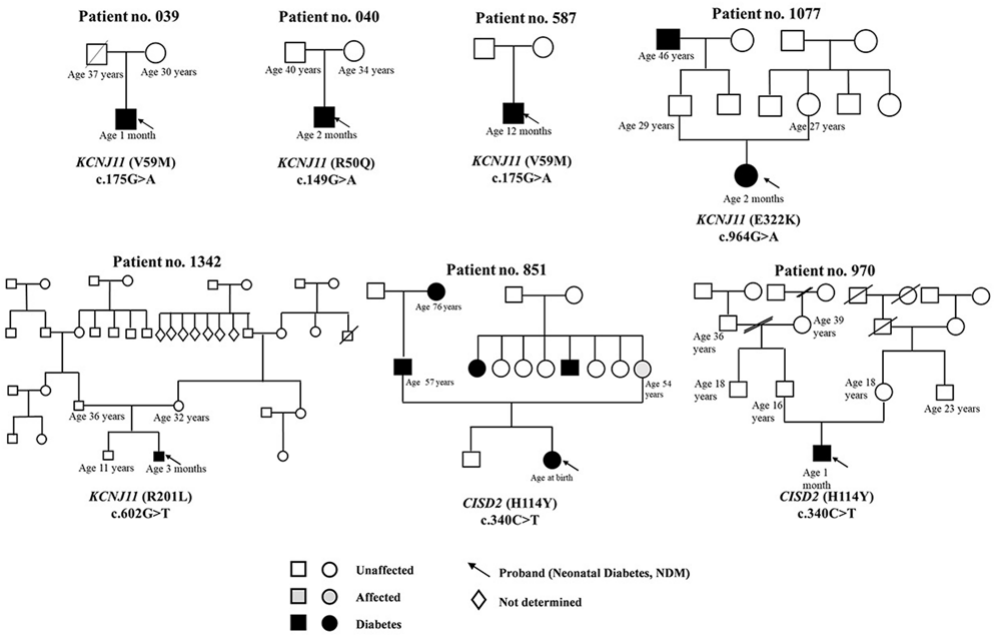
Family pedigrees of 7 patients with neonatal diabetes mellitus carrying variants in KCNJ11 (encoding the ATP-sensitive potassium channel subunit Kir6.2) and CISD2 (encoding CDGSH iron–sulfur domain-containing protein 2). Black symbols indicate patients with diabetes. Grey symbols indicate affected individuals. Clear symbols indicate unaffected individuals. Symbols marked with an arrow denote the proband (neonatal diabetes, NDM)

Four distinct *KCNJ11* variants were identified in five of the 14 patients (36%), and one *ABCC8* variant was detected (Fig. 2). All *KCNJ11* variants were classified as pathogenic or likely pathogenic in the ClinVar database, except for rs80356624 (chr11: 17409037, c.602G > T, p.Arg201Leu), which was not classified (Fig. 3). The *ABCC8* missense variant rs80356640 (chr11: 17483325, c.627 C > A, p.Asp209Glu) also had no pathogenicity status in ClinVar. In addition, two pathogenic *INS* variants—one in the promoter region (rs748749585, chr11: 2182532, c.-332 C > G) and one in the intronic region (rs797045623, chr11: 2181258 c.188-31G > A)—were detected in identical twins.

Additional variants in *WFS1*,* GATA6*,* DOCK8*,* COQ2*,* CISD2/SLC9B1*,* LRBA*, and *EIF2AK3* were identified in the remaining patients (Table 2). One individual with a homozygous *EIF2AK3* variant (rs1205989324, c.1650 + 1G > A) was diagnosed with Wolcott‒Rallison syndrome and presented with developmental dysplasia of the hip (Supplement Fig. 2). ClinVar categorizes this variant as likely pathogenic. Both parents and the patient’s younger brother carried the variant in a heterozygous state; only the mother had diabetes, diagnosed at age 35. An older brother with normal glucose tolerance did not carry the variant. Variants detected in *WFS1*,* GATA6*,* DOCK8*, and *LRBA* were documented in ClinVar as benign, likely benign, or of uncertain significance, while those in *COQ2* and *CISD2/SLC9B1* were not reported in ClinVar. All identified variants are summarized in Table 2. The overall diagnostic yield of WES based on previously reported Pathogenic and Likely Pathogenic variants in our cohort was 57% (8/14 patients).

**Table 2:** Identified genetic variants in Thai patients with neonatal diabetes mellitus

Patient number	Gene	Chr: Position GRCh37/hg19	dbSNP	Allele	Nucleotide change/Protein change	Type of variant	Amino Acid Conservation	Pathogenicity ClinVar	Classification ACMG	Clinical characteristics
039	*KCNJ11*	Chr11: 17,409,464	rs80356616	C > T	p.Val59Met	Missense	Yes	ClinVar ID: 8667 (Pathogenic)	Pathogenic	DEND syndrome Hypoglycemia Severe intellectual disability Short stature Global delay development with FTT with microcephaly Acute gastritis with moderate dehydration
040	*KCNJ11*	Chr11: 17,409,490	rs80356611	C > T	p.Arg50Gln	Missense	Yes	ClinVar ID: 36,431 (Pathogenic/Likely Pathogenic)	Pathogenic	Transient neonatal diabetes Glibenclamide treatment
133	*ABCC8*	Chr1: 17,483,325	rs80356640	C > A	p.Asp209Glu	Missense	Yes	ClinVar ID: 21,168 (Not provided)	Likely pathogenic	Diabetes
587	*KCNJ11*	Chr11: 17,409,464	rs80356616	C > T	p.Val59Met	Missense	Yes	ClinVar ID: 8667 (Pathogenic)	Pathogenic	Diabetes
599* and 600*	*INS*	Chr11: 2,182,532	rs748749585	C > G	c. -332	Promotor	-	ClinVar ID: 431,443 (Pathogenic)	Pathogenic	Diabetes
	*INS*	Chr11: 2,181,258	rs797045623	G > A	c.188 − 31	Intronic	-	ClinVar ID: 211,186 (Pathogenic)	Pathogenic	Diabetes
1077	*KCNJ11*	Chr11: 17,408,675	rs193929355	C > T	p.Glu322Lys	Missense	Yes	ClinVar ID: 21,203 (Pathogenic)	Pathogenic	Hyperglycemic/Hyperosmolar state
1342	*KCNJ11*	Chr11: 17,409,037	rs80356624	G > T	p.Arg201Leu	Missense	Yes	ClinVar ID: 21,199 (Not provided)	Pathogenic	-
015	*LRBA*	Chr4: 151,827,481	rs776254567	C > T	p.Gly524Ser	Missense	Yes	ClinVar ID: 726,390 (Likely benign)	Pathogenic	Diabetes Combined immunodeficiency
187	*EIF2AK3*	Chr2: 88,885,358	rs1205989324	G > A	c.1650 + 1	Splice donor	-	ClinVar ID: 445,635 (Likely pathogenic)	Pathogenic	Wolcott-Rallison syndrome Developmental dysplasia of the hip Short stature Beta thalassemia trait Cataract CKD stage II Aortic regurgitation and Mitral regurgitation is suspected due to rheumatic heart disease
579	*DOCK8*	Chr9: 286,593	rs529208	C > A	p.Pro97Thr	Missense	No	ClinVar ID: 178,766 (Benign)	Uncertain significance	Diabetes
599* and 600*	*WFS1*	Chr4: 6,303,248	rs1805069	G > A	p.Gly576Ser	Missense	No	ClinVar ID: 45,439 (Conflicting classifications of pathogenicity Uncertain risk allele; Benign; Likely benign)	Uncertain significance	Diabetes
	*GATA6*	Chr18: 19,751,292	rs559705145	C > T	p.Pro63Ser	Missense	No	ClinVar ID: 1,061,620 (Uncertain significance)	Uncertain significance	Diabetes
851	*CISD2/ SLC9B1*	Chr4: 103,808,519	rs201294540	C > T	p.His114Tyr	*CISD2*: Missense *SLC9B1*: Intronic	Yes	Not reported	Uncertain significance	Diabetes
	*WFS1*	Chr4: 6,302,889	rs1801208	G > A	p.Arg456His	Missense	No	ClinVar ID: 45,434 (Conflicting classifications of pathogenicity Uncertain significance; Benign; Likely benign)	Uncertain significance	Diabetes
970	*COQ2*	Chr4: 84,194,671	-	G > C	p.Leu224Val	Missense	No	Not reported	Uncertain significance	Diabetes
	*CISD2/SLC9B1*	Chr4: 103,808,519	rs201294540	C > T	p.His114Tyr	*CISD2*: Missense *SLC9B1*: Intronic	Yes	Not reported	Uncertain significance	Diabetes Small for gestational age Patent ductus arteriosus
	*WFS1*	Chr4: 6,302,889	rs1801208	G > A	p.Arg456His	Missense	No	ClinVar ID: 45,434 (Conflicting classifications of pathogenicity Uncertain significance; Benign; Likely benign)	Uncertain significance	Diabetes
1370	*WFS1*	Chr4: 6,302,889	rs1801208	G > A	p.Arg456His	Missense	No	ClinVar ID: 45,434 (Conflicting classifications of pathogenicity Uncertain significance; Benign; Likely benign)	Uncertain significance	Diabetes

*Genetic testing for identical twins (patients 599 and 600) was performed at the Molecular Genetics Laboratory, University of Exeter, United Kingdom

### Evidence of precision medicine in Thai NDM with KCNJ11 variants

**Fig. 4:**
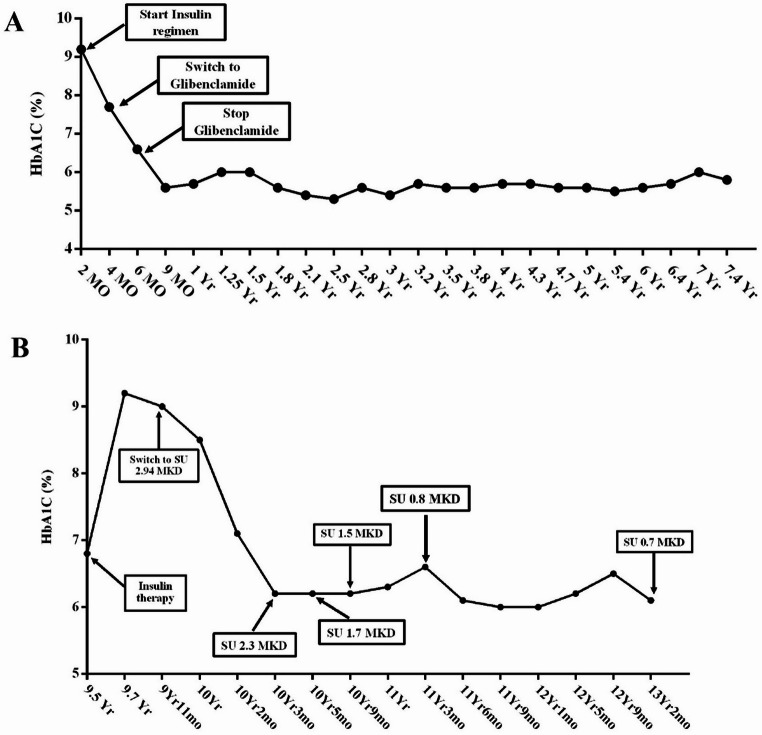
Timeline of glycemic control (HbA1c, %) in two patients with neonatal diabetes carrying *KCNJ11* variants, before and after transition from insulin to sulfonylurea (SU) therapy. **A** Patient no. 040 (c.149G > A, p.R50Q): initially treated with insulin, subsequently switched to glibenclamide, and later discontinued glibenclamide, achieving stable glycemic control. **B** Patient no. 039 (c.175G > A, p.V59M): initially treated with insulin, later transitioned to different doses of SU (0.7–2.94 mg/kg/day), achieving sustained glycemic control during long-term follow-up

NDM patients with *KCNJ11* or *ABCC8* variants exhibited a marked response to sulfonylurea therapy. Their glycated hemoglobin levels decreased significantly, and insulin dosages were reduced (Fig. 4). Table 3 highlights the characteristics of patients carrying these variants. In some instances, insulin and sulfonylurea treatments were discontinued. However, developmental delays remained unchanged at the follow-up six years after enrollment, showing no apparent improvement.

**Table 3:** Profiles of Thai patients with neonatal diabetes mellitus and *KCNJ11* or *ABCC8* mutations and hemoglobin A1c (HbA1c) outcomes during treatment

Patient number	KCNJ11 mutations	Age at diagnosis	Family history of DM	Maternal history of DM	Consanguineous marriage	DEND syndrome	HbA1c before SU treatment	HbA1c after SU treatment	Remarks
039	p.Val59Met (rs80356616)	1 month	No	No	No	Yes	8%	6.7%	Severe intellectual disability Short stature
040	p.Arg50Gln (rs80356611)	2 months	No	No	No	No	9.8%	5.8%	Insulin treatment was discontinued. SU treatment was stopped at the age of 6 months. No DEND syndrome.
587	p.Val59Met (rs80356616)	6 months	No	NA	NA	NA	13.1%	NA	-
1077	p.Glu322Lys (rs193929355)	2 months	Yes	NA	NA	NA	11.1%	NA	Hyperglycemic/Hyperosmolar state
1342	p.Arg201Leu (rs80356624)	3 months	No	NA	NA	NA	8.7%	NA	Moderate DKA
Patient number	*ABCC8* mutation	Age at diagnosis	Family history of DM	Maternal history of DM	Consanguineous marriage	DEND syndrome	HbA1c before SU treatment	HbA1c after SU treatment	Remarks
133	p.Asp209Glu (rs80356640)	6 months	-	-	NA	-	9.3%	7.0-7.5%	Developmental Delay-Continue treatment with glipizide and premixed insulin

*NA* Not available

## Discussion

NDM is a monogenic subtype of diabetes caused by variants in genes that regulate β-cell development and function (Beltrand et al. 2020). Many such genes have been identified, and their variants can cause hyperglycemia, influence extra-pancreatic manifestations, and alter treatment responses. Genetic heterogeneity thus contributes to the diverse phenotypes observed in NDM.

In our study, *KCNJ11* variants were the most common cause of NDM, accounting for 36% of cases (*n* = 14). Notably, *KCNJ11* and *ABCC8* encode the Kir6.2 and SUR1 subunits, respectively, of the pancreatic β-cell ATP-sensitive potassium (KATP) channel. These two genes are the most frequent causes of both transient and permanent NDM. Variants in *KCNJ11* or *ABCC8* impair the KATP channel’s sensitivity to ATP, causing persistent channel opening, membrane hyperpolarization, and reduced insulin secretion (Ashcroft 2007). High-dose sulfonylurea therapy addresses this defect by effectively closing the channel (Nichols et al. 2022), leading to improved glycemic control.

Our patients harboring *KCNJ11* variants (c.149G > A; p.Arg50Gln and c.175G > A; p.Val59Met) successfully transitioned from insulin to oral sulfonylurea therapy, achieving stable fasting glucose levels, in agreement with earlier reports. Since Kir6.2 is expressed in skeletal muscle, peripheral nerves, and the brain (Hattersley and Ashcroft 2005), specific *KCNJ11* variants are associated with developmental delay and epilepsy, collectively known as developmental delay, epilepsy, and NDM (DEND) syndrome (Flanagan et al. 2006). One patient carrying the p.Val59Met (c.175G > A) variant had NDM and developmental delay but no epilepsy, consistent with an intermediate DEND phenotype. Previous studies have shown improved glycemic control and neurological outcomes with early sulfonylurea therapy in patients harboring the p.Val59Met substitution (Chan and Laffel 2007; Pearson et al. 2006; Prado-Carro et al. 2014). However, our patient did not exhibit neurological improvement, likely due to the later initiation of sulfonylurea and additional factors—such as nutrition, family support, socioeconomic circumstances, and specialized training programs—that influence neurodevelopment (Bliznashka et al. 2024; Nguyen et al. 2018). Our findings illustrate that the same *KCNJ11* variant does not always present with identical clinical features. Indeed, different loci within *KCNJ11* correlate with variable severity of disease manifestations (Proks et al. 2004). Despite this variability, sulfonylurea remains the therapy of choice for patients with *KCNJ11* or *ABCC8* variants. More than 400 such patients have successfully transitioned from insulin to sulfonylurea, achieving better glycemic control and experiencing fewer hypoglycemic episodes (Pearson et al. 2006).

We identified a homozygous *EIF2AK3* variant (c.1650 + 1G > A, rs1205989324) in a patient with Wolcott‒Rallison syndrome (OMIM 226980) and developmental dysplasia of the hip. His mother, who had diabetes, and his younger brother carried the same heterozygous variant but showed no Wolcott‒Rallison syndrome features. This observation supports an autosomal recessive, splice-site defect of this variant in *EIF2AK3* and its association with disease manifestation.

We also detected variants in several genes previously linked to NDM. A missense *CISD2/SLC9B1* variant (c.340 C > T; p.His114Tyr) was found in two unrelated probands but was not listed in ClinVar. The affected amino acid position (p.His114Tyr) of c.340 C > T (rs201294540) is conserved across species (Supplemental Fig. 1). Histidine is an electrically charged side chain, whereas tyrosine is uncharged and polar. Additionally, this variant was classified as being of uncertain significance according to the ACMG classification (Supplemental Table 1). *CISD2* encodes a zinc finger protein located in the endoplasmic reticulum (ER) and the outer mitochondrial membrane. This variant alters the CDGSH iron‒sulfur domain-containing protein 2, and previous findings indicate that defective *CISD2* increases calcium flux from the ER to mitochondria, causing cytosolic calcium abnormalities in patient-derived fibroblasts. The resultant calcium imbalance is associated with augmented ER–mitochondria contact, a distended ER lumen, and a hyperfused mitochondrial network, all in the absence of overt ER stress. Moreover, culturing patient fibroblasts in glucose-free galactose medium revealed deficiencies in respiratory chain complexes I and II, with marginally reduced ATP levels (Rouzier et al. 2017). Because normal calcium homeostasis and mitochondrial function are crucial for insulin secretion, this variant likely impairs insulin release, leading to diabetes. Functional studies using an appropriate model are therefore warranted. *CISD2* is expressed in multiple tissues, including the pancreas and heart, which may explain the combination of patent ductus arteriosus, small-for-gestational-age status, and diabetes in this patient.

Other variants in *LRBA*,* DOCK8*,* WFS1*,* GATA6*, and *COQ2* were classified as benign, likely benign, or of uncertain significance, or were not documented in ClinVar. Because most of these amino acid changes were not located in conserved regions (except for *LRBA*), their pathogenic potential is unclear. Our study identified three variants (*WFS1*: rs1805069 (c.1726G > A; p.Gly576Ser) and rs1801208 (c.1367G > A; p.Arg456His), *GATA6*: rs559705145 (c.187 C > T; p.Pro63Ser) that are similar to those found in Thai T1D patients with negative autoantibodies (Teerawattanapong et al. 2024). Nevertheless, patients harboring these variants are valuable for further genetic investigations. WES (rather than targeted sequencing) may help uncover novel NDM-associated variants in the Thai population.

This study represents the first molecular genetics investigation of NDM in Thailand, a Southeast Asian country. Our findings demonstrate that *KCNJ11* variants are a major cause of NDM in this population. They also support sulfonylurea therapy as the treatment of choice for NDM caused by *KCNJ11* variants, underlining the importance of molecular genetics in precision diabetes care. We additionally identified a novel *CISD2/SLC9B1* variant in two unrelated NDM probands.

Notably, we could not identify causative genes in some patients, emphasizing the need to discover other NDM-associated genes in Thai populations. Our study was limited by a small sample size, incomplete treatment data, and incomplete phenotypic follow-up. Because the work was conducted at a single tertiary care university hospital, selection bias cannot be ruled out. Moreover, our WES analytical approach prevented the identification of novel genes. Larger-scale genomic studies are warranted to further elucidate the genetic landscape of NDM in Thailand and to inform more targeted treatment strategies. The lack of pathogenic variants in some patients may reflect the limitations of WES, which does not reliably capture structural variants, deep intronic changes, or regulatory region variants. In addition, novel NDM-associated genes that have not yet been characterized may account for unsolved cases. Whole-genome sequencing or functional studies may therefore be required to fully elucidate the genetic causes of NDM.

## Conclusion

WES identified *KCNJ11* variants as a primary cause of NDM in Thai patients. Individuals carrying these variants responded remarkably well to sulfonylurea therapy, highlighting an example of precision medicine in diabetes care. Larger-scale studies employing non-WES are needed to reveal novel NDM-related genes and provide more accurate prevalence estimates. Such discoveries will enhance our understanding of NDM pathogenesis and potentially lead to innovative therapeutic strategies.

## Supplementary Information

Below is the link to the electronic supplementary material.

Supplementary Material 1

## Data Availability

Data are available upon reasonable request.

## Abbreviations

DEND: Developmental delay, epilepsy and neonatal diabetes
ER: Endoplasmic Reticulum
NDM: Neonatal diabetes mellitus
WES: Whole Exome Sequencing
