# Supplementary material for: Targeted analysis of whole exome sequencing in Thai patients with neonatal diabetes
Source: Hum Genet. 2026 Jan 7;145(1):11. doi: 10.1007/s00439-025-02815-0 (PMC12779698; doi:10.1007/s00439-025-02815-0)

**Supplementary Figure 1.** Conserved protein sequence alignment of identified variants in patients with neonatal diabetes, generated using the NCBI Multiple Sequence Alignment Viewer (Version 1.25.2).

**NW133**: *ABCC8* (p.Asp209Glu)


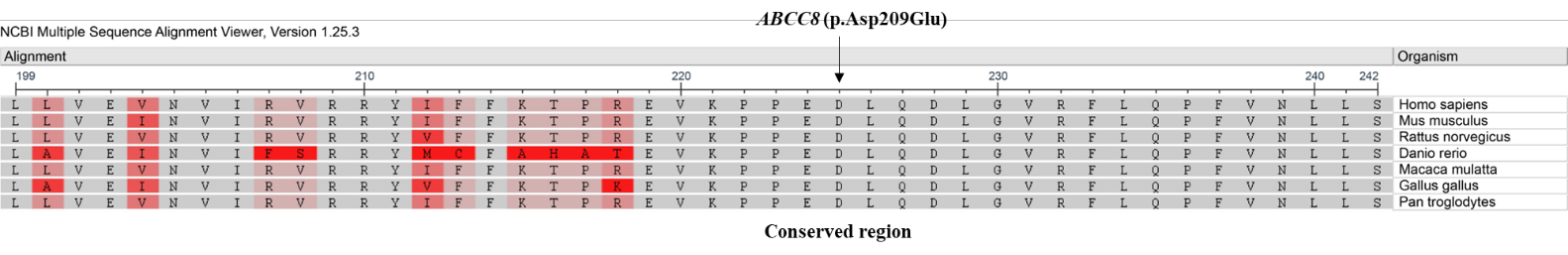


**NW039 and NW 587**: *KCNJ11* (p.Val59Met)

**NW040**: *KCNJ11* (p.Arg50Gln)


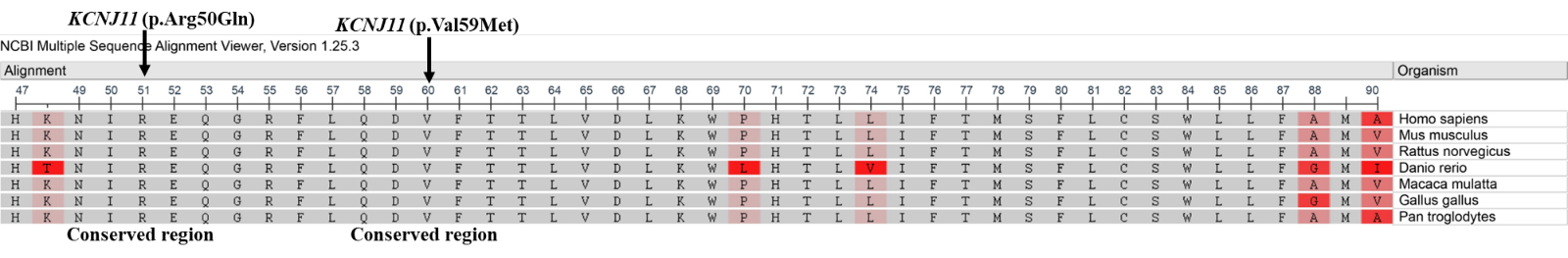


**NW1342**: *KCNJ11* (p.Arg201Leu)


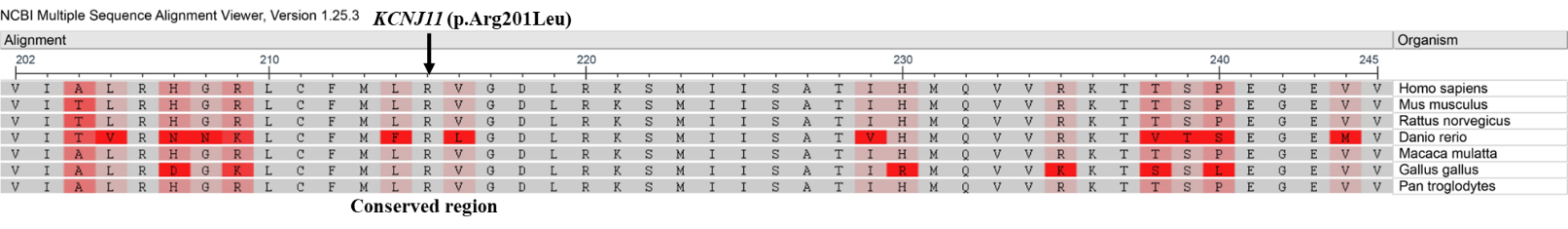


**NW1077**: *KCNJ11* (p.Glu322Lys)


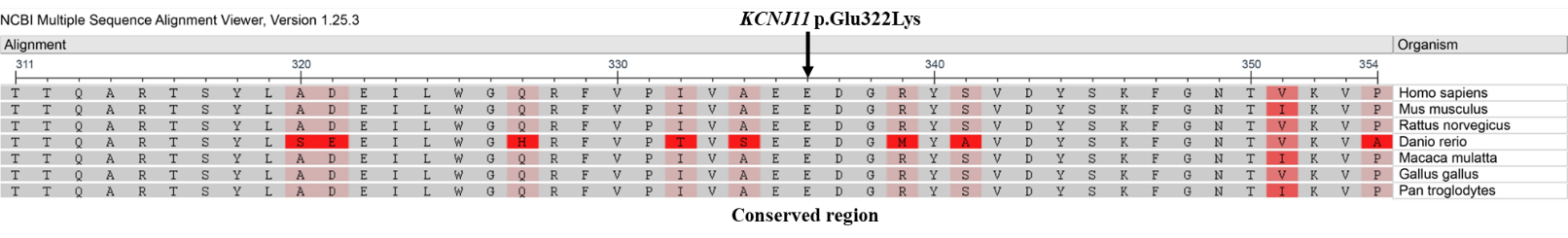


**NW015:** *LRBA* (p. Gly524Ser)


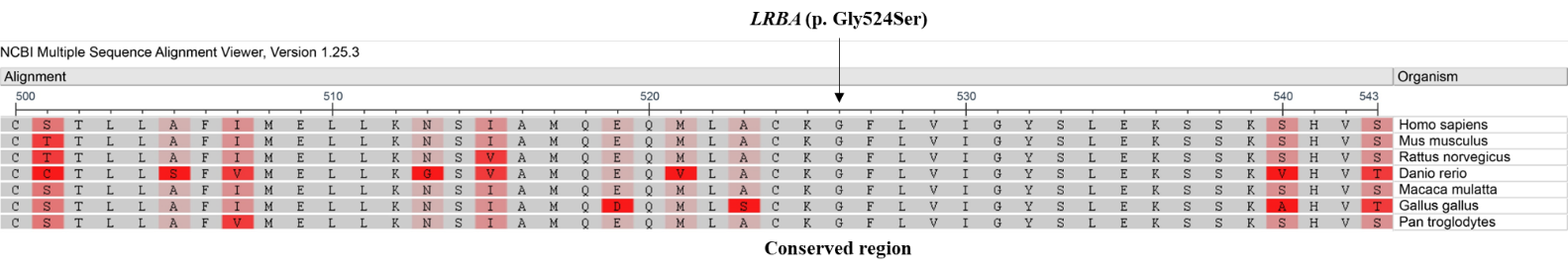


**NW579:** *DOCK8* (p. Pro97Thr)


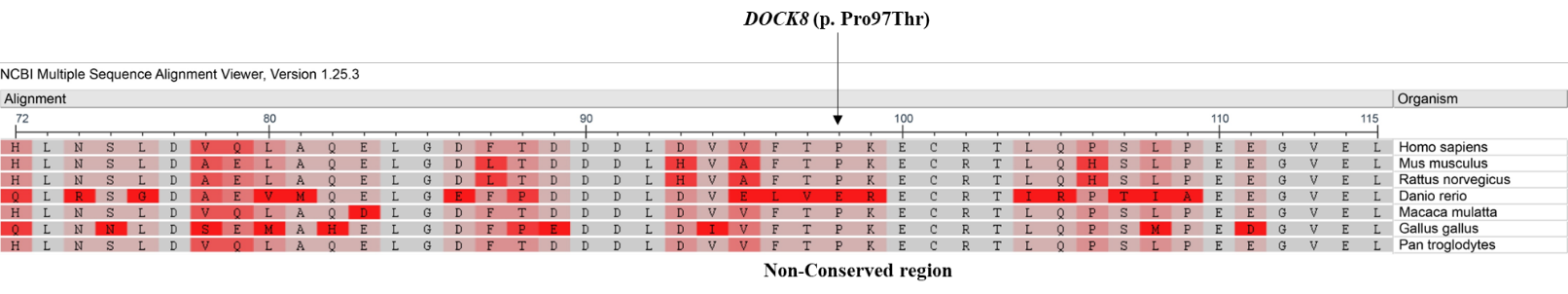


**NW599 and NW600:** *WFS1* (p. Gly576Ser)

**
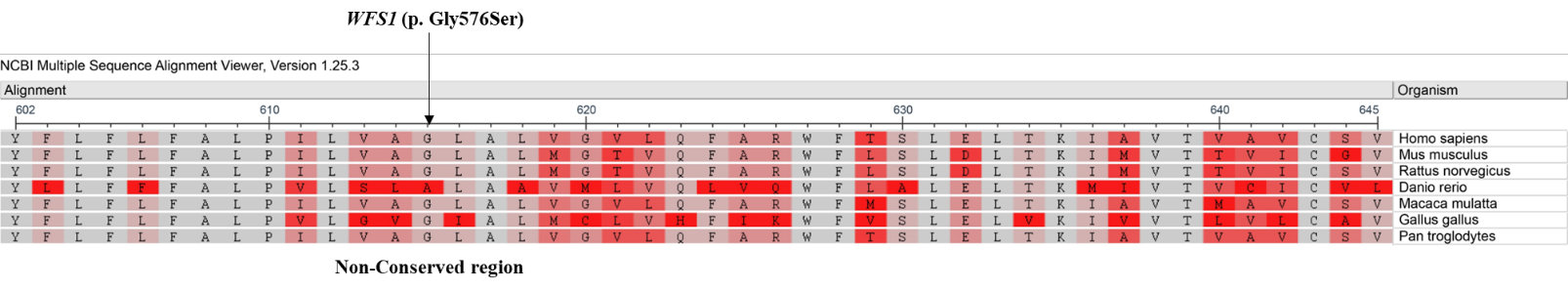
**

**NW599 and NW600:** *GATA6* (p. Pro63Ser)

**
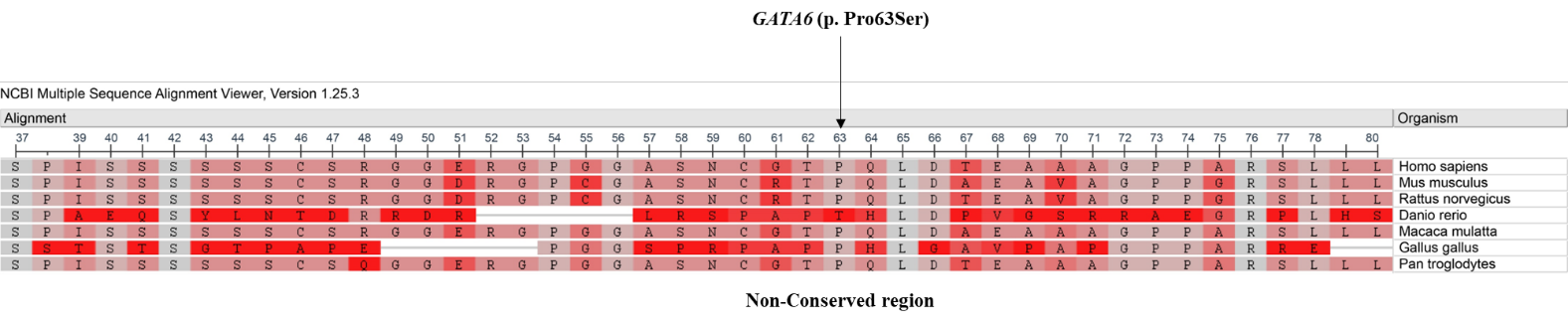
**

**NW851 and NW1370:** *WFS1* (p. Arg456His)


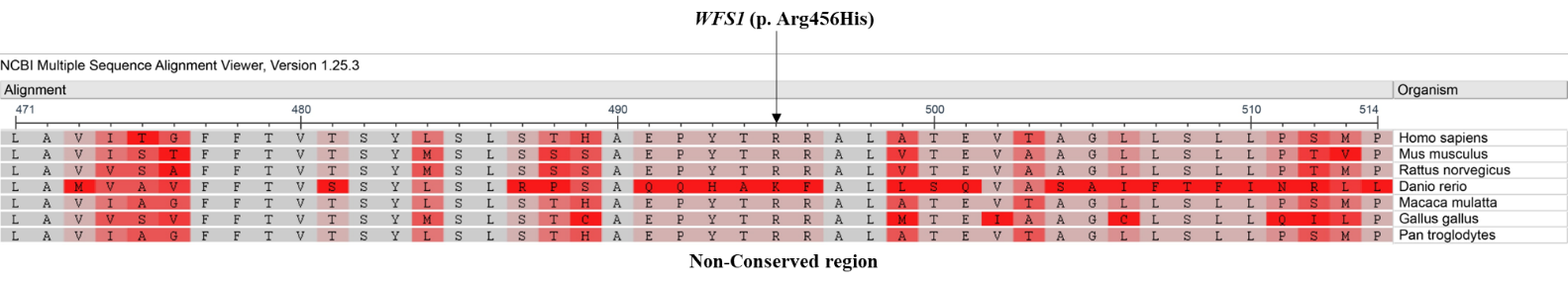


**NW851 and NW970:** *CISD2* (p.His114Tyr)


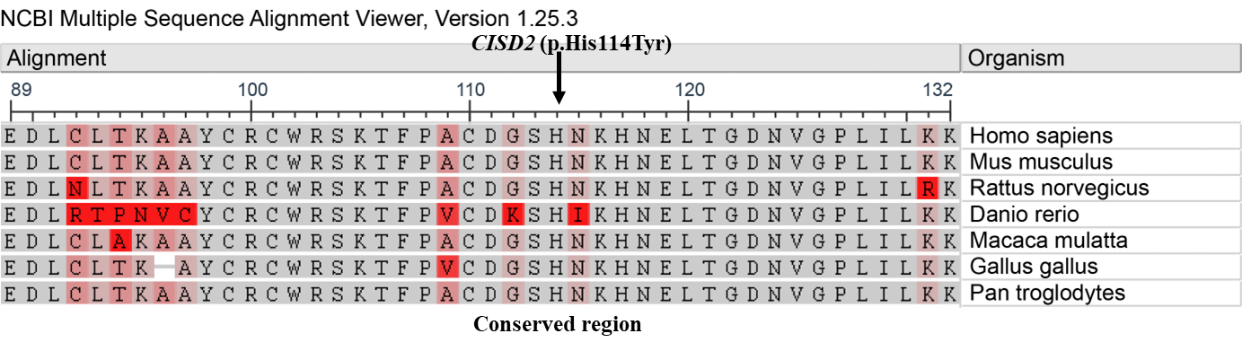


**NW970:** *COQ2* (p.Leu224Val)


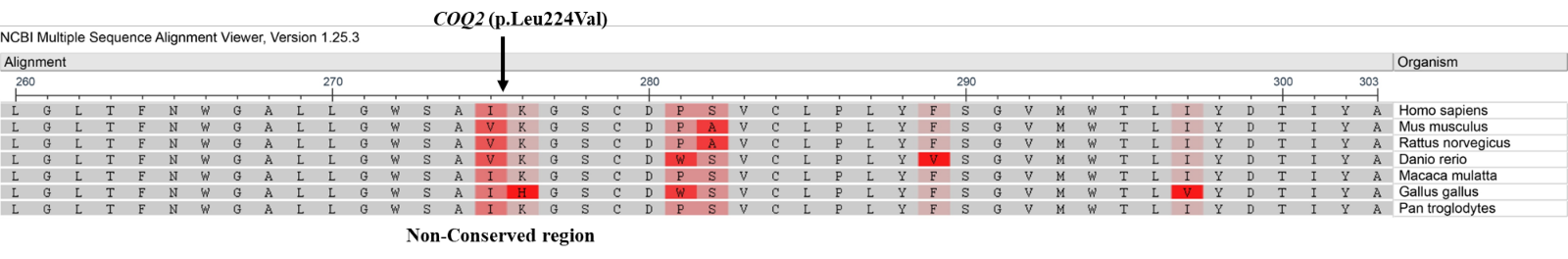


**Supplementary Figure 2** Pedigree of a patient with Wolcott–Rallison syndrome due to a homozygous *EIF2AK3* splice-site variant (rs1205989324, c.1650+1G>A). Black symbols indicate patients with diabetes. Clear symbols indicate unaffected individuals. Symbols marked with an arrow denote the proband (neonatal diabetes, NDM).


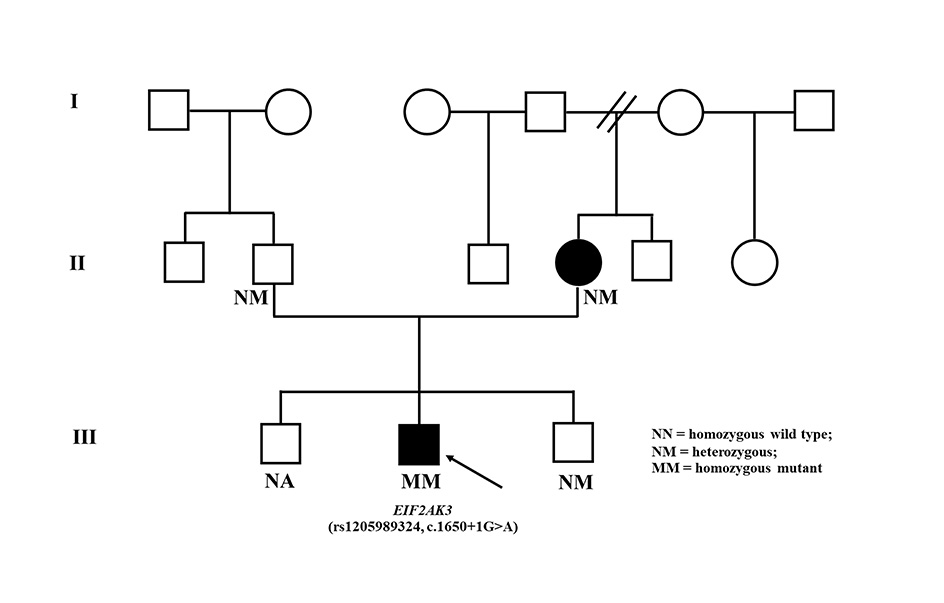

Supplement: Supplementary file 1 — Supplementary Material 1 [file 439_2025_2815_MOESM1_ESM.docx]
